# Bacterial Disease and Antimicrobial Susceptibility Patterns in HIV-Infected, Hospitalized Children: A Retrospective Cohort Study

**DOI:** 10.1371/journal.pone.0003260

**Published:** 2008-09-24

**Authors:** Heather B. Jaspan, Lyen C. Huang, Mark F. Cotton, Andrew Whitelaw, Landon Myer

**Affiliations:** 1 University of Cape Town School of Child and Adolescent Health, Cape Town, South Africa; 2 University of Cape Town School of Public Health and Family Medicine, Cape Town, South Africa; 3 Pediatric Infectious Disease Unit, Department of Pediatrics and Child Health, Stellenbosch University, Cape Town, South Africa; 4 National Health Laboratory Service, Division of Medical Microbiology University of Cape Town, Cape Town, South Africa; 5 Department of Epidemiology, Mailman School of Public Health, Columbia University, New York, New York, United States of America; Columbia University, United States of America

## Abstract

**Background:**

Serious bacterial infections are a major source of morbidity and mortality in HIV-infected children. The spectrum of disease is wide, and responsible organisms vary according to setting. The use of antibiotic prophylaxis and the emergence of multi-drug resistant bacteria necessitate examination of responsible organisms and their antibiotic susceptibility.

**Methodology/Principal Findings:**

A retrospective cohort study of all HIV-positive pediatric admissions at an urban public sector hospital in Cape Town between January 2002 and June 2006 was conducted. Children between the ages of one month and nine years with laboratory confirmed HIV status, serious bacterial infection, and a hospital length of stay of 5 days or more, were eligible for inclusion. Organisms isolated from blood, urine, and cerebral spinal fluid cultures and their antimicrobial susceptibility were examined, and compared according to timing of isolation to distinguish nosocomial versus community-acquired. One hundred and forty-one children were identified (median age 1.2 years), 39% of whom were on antiretrovirals started before or during this hospitalization. Bacterial infections involved all organ systems, however pneumonia was most common (67%). *S. pneumoniae* and *S. aureus* were the most common gram positive and *K. pneumoniae* was the most common gram negative organism. *K pneumoniae* isolates were resistant to many first and second line antibiotics, and were all considered nosocomial. All *S. aureus* isolates were methicillin resistant, some of which were community-acquired.

**Conclusions/Significance:**

Bacterial infections are an important source of co-morbidity in HIV-infected children in resource-limited settings. Clinicians should have a low threshold to initiate antibiotics in children requiring hospitalization. Broad-spectrum antibiotics should be used judiciously. Clinicians caring for HIV-infected children should be cognizant of the most common organisms affecting such children, and of their local antimicrobial susceptibilities, when treating empirically for serious bacterial infections.

## Introduction

Bacterial infections are a major source of morbidity and mortality in HIV- infected children, causing . a wide spectrum of diseases, many of which are included in the WHO and CDC staging systems[1], [2] In an early USA study predating both trimethoprim-sulfamethoxazole (TMP-SMX) and antiretroviral therapy (ART)[3], there were 160 episodes of minor and 48 serious bacterial infections (SBI) per 100 patient-years in HIV-infected children, demonstrating the extremely high burden of bacterial morbidity. A similar study performed after the introduction of TMP-SMX and zidovudine monotherapy documented a two-year SBI rate of 45% among children receiving TMP-SMX.[4]. In a randomized controlled trial of TMP-SMX prophylaxis for HIV-infected children in Zambia, the SBI admission rate was 19 per 100 child-years in the TMP-SMX group and 29 in the placebo group (P = 0.09), and the cumulative two-year probability of dying in hospital from SBI (predominantly pneumonia) was 7% on TMP-SMX and 12% on placebo (P = 0.08).[5] Thus, data from varied settings documented high rates of SBI in children and a protective effect of TMP-SMX.

Since the widespread availability of antiretroviral therapy (ART), there has been a marked decrease in the morbidity and mortality of bacterial infections in both resource rich and resource limited settings [6], [7]. In the USA, pneumonia is still the most common bacterial infection in HIV-infected children on ART, however, in comparison with the pr-ART era, the incidence has decreased from 11 to 2 events per 100 person years.[8] In a Californian cohort, bacterial infections accounted for 60 hospitalizations for 64 children in 1994, versus only 8 for 101 children in 2001.[9] The hospitalization rate for SBI was only 14.2 per 100 person years for a Thai cohort of children on ART.[10] Nevertheless, bacterial infections remained the most common reason for hospitalization.

HIV-infected children have a greater risk of bacterial infections than their HIV-negative counterparts[11], and these infections are more invasive, more likely to disseminate, and have worse outcome in HIV-infected children.[11]–[13] Also, HIV-infected children often have multiple diagnoses and polymicrobial infections.[11], [12] All organ systems can be involved by bacterial infections, and concomitant bacteraemia is common. Abscess formation in internal organs and skin can also occur. Otitis media (OM), acute sinusitis and mastoiditis are also common. By far the most frequent cause of bacterial morbidity in all HIV-infected children is pneumonia, both with and without TMP-SMX prophylaxis.[5]

The emergence of antimicrobial resistant organisms is a global problem, not restricted to children with HIV. However, the widespread use of TMP-SMX for prophylaxis may exacerbate antimicrobial resistance. In a South African study, only four of 26 *S. pneumoniae* from HIV-infected children with community acquired pneumonia were sensitive to TMP-SMX.[11] Resistance was unaffected by TMP-SMX prophylaxis, arguing that resistance may be firmly established in the community. The antimicrobial resistance patterns from blood cultures from HIV infected and uninfected children have been compared in only a few settings. The resistance of *both S. pneumoniae* and *S. aureus* to TMP-SMX was significantly higher in the HIV-infected group's isolates in Soweto [14]. In three African studies, almost 15% of the isolates from HIV-infected children were multiply resistant *E. coli* or Methicillin resistant *staphylococcus aureus* (MRSA). [14]–[16] However, in the Ugandan children, organisms from HIV-infected children were either more sensitive or had the same profile as uninfected children.[16] Therefore it is unclear how widespread antimicrobial resistance is in HIV-infected children.

## Methods

### Objectives

We sought to evaluate the spectrum of bacterial infection, causative organisms, and antibiotic resistance patterns of an inpatient cohort of HIV-infected children.

### Participants

We conducted a retrospective cohort study of all HIV-positive pediatric admissions to a Pediatric ward in an urban public sector hospital in Cape Town between January 2002 and June 2006. Over 50% of all admissions are HIV-infected. The referral population is a largely black African population living in rapidly growing settlements with residents of predominantly low socioeconomic status. Data were collected from paper charts and computer records. Where more than one hospitalization per patient occurred, only the first was examined.

Children between one month and nine years of age with laboratory confirmed HIV-infection, SBI and hospitalization for 5 or more days, were eligible for inclusion.

### Description of investigations

The following were considered SBI: bacterial sepsis, bacterial pneumonia, urinary tract infection, and/or meningitis. For bacterial sepsis, children had organisms identified on blood culture. Diagnosis of bacterial pneumonia was made by clinical findings, laboratory results including high white blood cell count with neutrophil predominance, lobar consolidation on chest radiograph, and blood cultures when available. For meningitis, either an organism was isolated from cerebrospinal fluid or the chemistry and cell counts were suggestive of bacterial infection. Coagulase negative staphylococcus only included as a pathogen if isolated from more than one culture or from a patient with indwelling invasive lines. All *S. aureus* isolates were considered pathogenic, as the patients were ill. An infection was considered community acquired if symptomatic at admission or if cultures taken at or before 48 hours after admission were positive. If positive cultures or new symptoms appeared after 48 hours, the infection was considered nosocomial or hospital acquired [17].

Clinical specimens submitted for bacterial culture were inoculated into appropriate media depending on the nature of the specimen. Organisms were identified by standard biochemical and/or serological methods. Antimicrobial susceptibility testing was done by the Kirby-Bauer disc diffusion technique, and MICs (if necessary) were performed using E-tests. Susceptibilities were not available for all specimens from 2002–2003. Therefore, susceptibilities represent more recent specimens.

### Ethics

Approval for this study was obtained from the University of Cape Town Faculty of Health Sciences Research Ethics Committee. Exemption from informed consent was granted as this was a retrospective study with no identifying data.

## Results

### Cohort Demographics

One hundred and forty-one children met inclusion criteria. The median age was 1.2 years (IQR 0.5–2.3) and 65 (46%) were female (table 1). The median weight on admission was 6.7 kg (IQR 4.7–9.6)), 16 (11.4%) with a diagnosis of marasmus and a further 27 (19%) labeled as failure to thrive.

**Table 1:** Demographics and spectrum of serious bacterial disease in hospitalized children with HIV.

Characteristic	n (%)
Gender	
Males	76 (54)
Females	65 (46)
On ART	55 (39)
On TB medication	39 (28)
Mortality	13 (9)
Age at admission (yrs)	1.2 (0.5–2.34)
Median (IQR)	
Weight at admission (kg)	6.7 (4.7–9.6)
Median (IQR)	
Temp. at admission (°C)	37.3 (36.5–38.3)
Median (IQR)	
Birth weight (kg)	2.8 (2.8–3.0)
Mean (95% CI)	
Hemoglobin on admission	9.1 (8.1–10.3)
Median (IQR)	
Concomitant Diagnoses	
TB	40 (28)
TB meningitis	2 (1)
Gastroenteritis	54 (38)
PEM	16 (11)
Fungal sepsis	4 (2.3)
Failure to thrive	29 (21)
Bacterial Infections	40 (28)
Bacterial sepsis	74 (52)
Pneumonia	95 (67)
% Bacteremic pneumonia	50
Bacterial meningitis	4 (3)
% Bacteremic meningitis	50
Urinary tract infection	15 (11)
% Bacteremic UTI	87
Skin or soft tissue, including abscess	2 (1.5)
Osteomyelitis or septic arthritis	1 (0.7)

ART–antiretroviral therapy, TB–Tuberculosis, PEM-Protein energy malnutrition, IQR–Interquartile range.

Approximately one third of children received ART(n = 55). However, the median time on ART was only 6 days (IQR -8–35), and many were started during this hospitalization. There were 40 children on tuberculosis treatment; some children were receiving both ART and TB treatment (n = 16; 11.3%). The median CD4 percentage was 17.2% (IQR 9.7–22.7), with a median absolute CD4 count of 509 cells/mm^3^ (IQR 244–759). The median hospital length of stay was 13 days (IQR 6–34 days). There were 13 deaths in hospital with the remainder being discharged.

### Spectrum of Disease

A lower respiratory tract infection was diagnosed in 95 (67%) of the children, while 74 (53%) had bacterial sepsis. The most prevalent bacterial infections in this cohort of hospitalized children were pneumonia and sepsis (Table 1). However, most other organ systems were affected, including the central nervous system (meningitis), lung, mastoid, bone and joints. A high proportion of patients with pneumonia, meningitis, and urinary tract infection had concomitant bacteremia. *Mycobacterium tuberculosis* was a frequent concomitant infection. Eight deaths had dual diagnoses of sepsis and pneumonia, and one had both sepsis and a UTI.

### Responsible Organisms

The relative frequencies of specific bacterial isolates from blood, urine and CSF cultures of HIV-infected children are shown in table 2 (excluding mycobacteria). The most common pathogen isolated from blood was *S. pneumoniae* followed by *S aureus*. The most common gram negative organism was *K. pneumoniae*. All *K. pneumoniae* isolates were from blood cultures taken more than two days into the admission, implying a nosocomial origin. In contrast, all but two *S pneumoniae* from blood culture were within two days of admission, therefore probably community acquired. The organisms most frequently present when the outcome was fatal were *S aureus* (n = 3, all MRSA), Coagulase negative staphylococcus (n = 3) and *K pneumoniae* (n = 3). Five of the 13 children who died grew multiple pathogenic organisms in blood or urine.

**Table 2:** Bacterial isolates from hospitalized HIV-infected children with serious bacterial infections*

	Blood n = 128 (%^1^)	Urine n = 21 (%^2^)	CSF n = 36 (%^2^)	N (%) assumed nosocomial (>48 hours)^3^
*S. pneumoniae*	21 (16)		1 (2.8)	2/22 (9%)
*S. aureus*	14 (11)			9/14 (64%)
Coagulase negative staphylococcus^4^	4 (3)			4/4 (100%)
*E. coli*	5 (4)	5 (24)		7/10 (70%)
*K. pneumoniae*	11 (9)	2 (9.5)		13/13 (100%)
Hemophilus spps	8 (7)		1 (2.8)	1/9 (11%)
*S. typhi*	1 (1)			0
Non typhoid salmonella	9 (7)	1 (4.8)		7/10 (70%)
*S. milleri*	1 (1)			1 (100%)
*Enterococcus faecalis*	4 (3)	2 (9.5)		5/6 (83%)
*Enterococcus faecium*	1 (1)			1 (100%)
*Enterobacter species*	6 (4)			4/6 (67%)
*P. aeruginosa*	1 (1)	1 (4.8)		0
Acinetobacter	4 (3)			3/4 (75%)

1 Percent of positive cultures, including fungal and mixed or skin flora

2 Percent of all cultures sent. Urine cultures were not done if urine dipstick was normal unless suspicion was high.

3 The proportion of nosocomial infections presented here reflects that from children hospitalized for five days or longer, and not necessarily the proportion of nosocomial infections from hospitalizations of all HIV-infected children.

4 Coagulase negative staphylococcus only included as a pathogen if isolated from more than one culture or with indwelling invasive lines.

### Susceptibility Patterns

The antimicrobial susceptibility patterns of each of the more prevalent organisms are shown in table 3. *S. pneumoniae* and *H. influenza* isolates were generally highly susceptible, whereas the majority of the *K. pneumoniae* isolates, most likely nosocomial, were highly resistant. All *K. pneumoniae* isolates were extended spectrum beta-lactamase producing; the only single antibiotic to which all *K. pneumoniae* isolates were susceptible was Meropenem. *S. aureus* whether community-acquired or nosocomial, was often resistant to TMP-SMX, and clindamycin, both frequently used antibiotics for resistant staphylococci. . Although community-acquired non-typhoid salmonella (NTS) were susceptible to first-line antibiotics such as ampicillin, the more frequently hospital-acquired organisms were more resistant. MRSA was isolated from a third of the blood cultures of children who died, as was *K. pneumoniae*, therefore resistant organisms were possibly responsible for a relatively high proportion of the mortality, and appropriate empiric therapy was not used.

**Table 3:** Antibiotic susceptibilities of the most common organisms isolated from blood cultures of children with serious bacterial infections, according to time of isolation.

% susceptible												
	*S. pneumoniae*		*S. aureus*		*H. influenzae*		*E. coli*		Non-typhoid salmonella		*K. pneumoniae*	
*Time* ( Total n)	*≤48 hrs* (14)	*>48 hrs* (2)	*≤48 hrs* (3)	*>48 hrs* (9)	*≤48 hrs* (3)	*>48 hrs* (2)	*≤48 hrs* (1)	*>48 hrs* (4)	*≤48 hrs* (3)	*>48 hrs* (6)	*≤48 hrs* (0)	*>48 hrs* (10)
Penicillin/Ampicillin	79^a^	50^a^	0	0	100	100	0	50	100	17	NA	0
Amoxicillin +clavulanate	NT	NT	NT	NT	100	100	100	50	100	100	NA	20^b^
TMP-SMX	NT	NT	0	0	67	0	0	75	100	17	NA	0
Cloxacillin/Methicillin	NT	NT	0	0	NT	NT	NT	NT	NT	NT	NT	NT
3^rd^ generation Cephalosporin	93^c^	100	NT	NT	100	100	100	50	100	20	NA	0
Erythromycin	86	50	0	0	NT	NT	NT	NT	NT	NT	NT	NT
Gentamicin	NT	NT	0	0	NT	NT	100	50	NT	NT	NA	10
Clindamycin	NT	NT	67	33	NT	NT	NT	NT	NT	NT	NT	NT
Ofloxacin/Ciprofloxacin	NT	NT	33	67	100	100	100	75	100	100	NA	40
Amikacin	NT	NT	100	78	NT	NT	100	100	NT	NT	NA	90^a^
Pipericillin/Tazobactam	NT	NT	NT	NT	NT	NT	100	75^a^,^c^	100	100	NA	50^a^
Meropenem	NT	NT	NT	NT	NT	NT	100	100	100	100	NA	100
Vancomycin	NT	NT	100	100	NT	NT	NT	NT	NT	NT	NT	NT

a The remainder were intermediate sensitivity

b Five isolates intermediate susceptibility

c One unknown

NT–not tested

NA–not applicable

TMP-SMX–Trimethoprim sulfamethoxazole

## Discussion

In this cohort of hospitalized HIV-infected children, pneumonia was the most common SBI resulting in hospitalization. There was frequent coincident bacteremia. This is consistent with other HIV-infected pediatric cohorts described from both developing and developed settings[5], [8]. The relative prevalence of most bacterial pathogens is similar to that of HIV-uninfected children. In a large Kenyan study *S. pneumoniae, H. influenzae*, NTS, and *E. coli* were more common in HIV-infected children [18]. These organisms were commonly seen in malnourished children in the pre-HIV era. *S. pneumoniae* is the most common organism in other cohorts, with *S. aureus*, *Salmonella* species, and *Enterobacteriaceae* occurring frequently [4], [11], [14], [15]. *P. aeruginosa* is also seen occasionally. The proportion of *H influenzae* is influenced by the introduction of the *H. influenzae* type B (Hib) vaccine, in 1999 in South Africa.

Bacteria that were assumed to be predominantly community acquired were generally susceptible to first line or narrow spectrum antibiotics (those that target generally only one class of bacteria). This included the *S. pneumoniae* isolates. In other settings, multi-drug resistant *S. pneumoniae* were increasing in frequency before introduction of the pneumococcal conjugate vaccine [19], [20]. In children with community acquired pneumonias in Soweto, South Africa, 50% of the *S. pneumoniae* isolates from the HIV-infected children were penicillin resistant versus 23% of the HIV-negative children's isolates.[14] The community-acquired *S. aureus* isolates were, however, all MRSA. The empiric treatment of community acquired bacterial infections in HIV-infected children in resource limited settings should bear in mind the antimicrobial susceptibility patterns of the region. Penicillin and ampicillin are effective against most penicillin-non-susceptible pneumococcal isolates in the case of isolated respiratory tract infections. The clinician must consider viral, fungal, and mycobacterial infections in the differential diagnosis of bacterial infections and treat accordingly.

The high proportion of *K. pneumoniae* resistant to third generation cephalosporin is highly concerning, particularly since all these infections were acquired after two days in hospital, and therefore probably hospital-acquired. Highly resistant organisms have been described from a similar setting [15], and is likely due to extended spectrum beta lactamase production. Since not all *K. pneumoniae* isolates were susceptible to piperacillin-tazobactam or amikacin, clinicians should consider empiric carbapenems for suspected hospital-acquired sepsis.

Nosocomial infections are more common in HIV-infected than in uninfected adults, with the most frequently isolated bacterial pathogens being *S. aureus and P. aeruginosa*, a high proportion of which are methicillin resistant [21]. Nosocomial infections in HIV-infected adults have a high mortality. There are few data on nosocomial bacterial infections in HIV-infected children. In a cohort of children hospitalized with TB, nosocomial infections were more common in HIV-infected patients, and more frequently fatal [22]. Although our data do not reflect the overall prevalence of nosocomial infections in hospitalized HIV-infected children due to our study design, the data may represent common resistance patterns of these organisms.

The most effective public health approach to improving infectious disease burden is vaccination. Effective vaccines are licensed for Hib and *S. pneumoniae*. Both are polysaccharide protein conjugate vaccines. The Hib vaccine is now widely available including in developing countries. Although the estimated efficacy of the Hib vaccine is decreased and the risk for vaccine failure increased in HIV-infected children,[23], [24] the introduction of Hib vaccine into countries with high HIV prevalence has greatly decreased Hib disease burden.[25] In 2000, a pneumococcal conjugate vaccine (PCV) was licensed in the USA. Although PCV is less immunogenic in HIV-infected children than non-infected children,[26] this also seems to improve in children receiving ART.[27] A similar 9-valent PCV has been extensively studied in South Africa, and similar efficacy is seen in these HIV-infected infants.[28] Unfortunately, PCV is not widely accessible to children in less resourced countries, and its access should become a global priority.

These data confirm that pneumonia is the most common SBI in HIV-infected children, but with a wide spectrum of presentation. The predominance of MRSA is alarming. The high proportion of nosocomially acquired highly resistant *K. pneumoniae,* suggest that infection control practices should be implemented to avoid the spread of antibiotic resistance. While overuse of broad-spectrum antibiotics should be avoided, judicious limited use is indicated for suspected nosocomial sepsis.

### Limitations

The limitations of these data are that they are retrospective and therefore incomplete. In addition, the isolation of organisms is dependant on adequate blood volumes and rapid transport to the microbiology laboratory, both of which may have been limited in the setting of sick, malnourished children in a resource-limited hospital. Our findings may not be generalizable to other better resourced settings such as those with more nursing staff, and less ward crowding, or where all HIV-infected children are on ART.
